# Supplementary material for: The Systems Biology Research Tool: evolvable open-source software
Source: BMC Syst Biol. 2008 Jun 29;2:55. doi: 10.1186/1752-0509-2-55 (PMC2446383; doi:10.1186/1752-0509-2-55)
Supplement: Additional file 1 — SBRT Archive. An archive of the current version of the Systems Biology Research Tool. [file 1752-0509-2-55-S1.zip › sbrt-1.4.0/doc/developers_guide/api/sbrt/kernel/math/algebra/SparseDoubleMatrixWrap.html]

SparseDoubleMatrixWrap


|  |  |  |
| --- | --- | --- |
| **Overview** \| **Package** \| **Class** \| **Use** \| **Tree** \| **Deprecated** \| **Index** \| **Help** | |  |
| **PREV CLASS**   **NEXT CLASS** | **FRAMES**    **NO FRAMES**     **All Classes** |  |
| SUMMARY: NESTED \| FIELD \| CONSTR \| METHOD | DETAIL: FIELD \| CONSTR \| METHOD |  |


---


## sbrt.kernel.math.algebra Class SparseDoubleMatrixWrap<R,C>

```
java.lang.Object
  sbrt.kernel.math.algebra.MatrixWrap<R,C,java.lang.Double>
      sbrt.kernel.math.algebra.DoubleMatrixWrap<R,C>
          sbrt.kernel.math.algebra.SparseDoubleMatrixWrap<R,C>
```

**Type Parameters:**: `R` - the row type.: `C` - the column type.

**All Implemented Interfaces:**: DoubleMatrix<R,C>, Matrix<R,C,java.lang.Double>, SparseDoubleMatrix<R,C>

---

``` public class SparseDoubleMatrixWrap<R,C> extends DoubleMatrixWrap<R,C> implements SparseDoubleMatrix<R,C> ```

This class is used to wrap mutable matrices as an immutable
`SparseDoubleMatrix`. This type of wrapping eliminates the possibility
of casting a mutable matrix back to its mutable type. This ensures that a
mutable matrix can be viewed by others in a safe way, without fear of
modification.

Instances of this class are immutable.

**Author:**
:   This class was written and documented by Jeremiah Wright while in the
    Wagner lab.

---

**Constructor Summary**

`SparseDoubleMatrixWrap(SparseDoubleMatrix<R,C> m)`
  
          Constructs a new wrapper around the provided matrix.


| **Method Summary** | |
| --- | --- |
| `java.util.Set<C>` | `getColIndNzEl(java.lang.Object rowIndex)`             Returns the set of column indices whose elements are nonzero in the row corresponding to the provided index. |
| `SparseDoubleMatrix<R,C>` | `getColumn(java.lang.Object columnIndex)`             Returns the column corresponding to the provided column index. |
| `SparseDoubleMatrix<R,C>` | `getRow(java.lang.Object rowIndex)`             Returns the row corresponding to the provided row index. |
| `java.util.Set<R>` | `getRowIndNzEl(java.lang.Object columnIndex)`             Returns the set of row indices whose elements are nonzero in the column corresponding to the provided index. |

**Methods inherited from class sbrt.kernel.math.algebra.DoubleMatrixWrap**

`getElement, toLinearCombs`

**Methods inherited from class sbrt.kernel.math.algebra.MatrixWrap**

`columns, equals, getColumnIndices, getRowIndices, hashCode, isColumnIndex, isEmpty, isRowIndex, rows, toString`

**Methods inherited from class java.lang.Object**

`clone, finalize, getClass, notify, notifyAll, wait, wait, wait`

**Methods inherited from interface sbrt.kernel.math.algebra.DoubleMatrix**

`getElement, toLinearCombs`

**Methods inherited from interface sbrt.kernel.math.algebra.Matrix**

`columns, equals, getColumnIndices, getRowIndices, hashCode, isColumnIndex, isEmpty, isRowIndex, rows, toString`

**Constructor Detail**

### SparseDoubleMatrixWrap

```
public SparseDoubleMatrixWrap(SparseDoubleMatrix<R,C> m)
```

:   Constructs a new wrapper around the provided matrix.

    **Parameters:**: `m` - the matrix to be wrapped by this wrapper object. **Throws:**: `java.lang.NullPointerException` - if the provided matrix is `null`.


**Method Detail**

### getRowIndNzEl

```
public java.util.Set<R> getRowIndNzEl(java.lang.Object columnIndex)
```

:   Returns the set of row indices whose elements are nonzero
    in the column corresponding to the provided index.

    :   **Specified by:**: `getRowIndNzEl` in interface `SparseDoubleMatrix<R,C>`
    :   **Parameters:**: `columnIndex` - the column index. **Returns:**: the set of row indices whose elements are nonzero in the column corresponding to the provided index.

---


### getColIndNzEl

```
public java.util.Set<C> getColIndNzEl(java.lang.Object rowIndex)
```

:   Returns the set of column indices whose elements are nonzero
    in the row corresponding to the provided index.

    :   **Specified by:**: `getColIndNzEl` in interface `SparseDoubleMatrix<R,C>`
    :   **Parameters:**: `rowIndex` - the row index. **Returns:**: the set of column indices whose elements are nonzero in the row corresponding to the provided index.

---


### getRow

```
public SparseDoubleMatrix<R,C> getRow(java.lang.Object rowIndex)
```

:   Returns the row corresponding to the provided row index.
    A row is itself a 1 x n matrix, where n is the number of
    columns in this matrix.

    :   **Specified by:**: `getRow` in interface `DoubleMatrix<R,C>` **Specified by:**: `getRow` in interface `Matrix<R,C,java.lang.Double>` **Specified by:**: `getRow` in interface `SparseDoubleMatrix<R,C>` **Overrides:**: `getRow` in class `DoubleMatrixWrap<R,C>`
    :   **Parameters:**: `rowIndex` - the index of the row to be returned. **Returns:**: the row corresponding to the provided row index.

---


### getColumn

```
public SparseDoubleMatrix<R,C> getColumn(java.lang.Object columnIndex)
```

:   Returns the column corresponding to the provided column index.
    A column is itself an m x 1 matrix, where m is the number of
    rows in this matrix.

    :   **Specified by:**: `getColumn` in interface `DoubleMatrix<R,C>` **Specified by:**: `getColumn` in interface `Matrix<R,C,java.lang.Double>` **Specified by:**: `getColumn` in interface `SparseDoubleMatrix<R,C>` **Overrides:**: `getColumn` in class `DoubleMatrixWrap<R,C>`
    :   **Parameters:**: `columnIndex` - the index of the column to be returned. **Returns:**: the column corresponding to the provided column index.


---


|  |  |  |
| --- | --- | --- |
| **Overview** \| **Package** \| **Class** \| **Use** \| **Tree** \| **Deprecated** \| **Index** \| **Help** | |  |
| **PREV CLASS**   **NEXT CLASS** | **FRAMES**    **NO FRAMES**     **All Classes** |  |
| SUMMARY: NESTED \| FIELD \| CONSTR \| METHOD | DETAIL: FIELD \| CONSTR \| METHOD |  |


---
